# YouTube Videos as Educational Resources for Seizure Clusters and Treatment Options: Cross-Sectional Observational Study

**DOI:** 10.2196/98768

**Published:** 2026-09-30

**Authors:** Taylor S Elliott, Andrew J Gienapp, James W Wheless

**Affiliations:** 1 College of Medicine, University of Tennessee Health Science Center, 910 Madison Ave, Memphis, TN, 38163, United States, 1 (901) 448-5500; 2 Comprehensive Epilepsy Program, Neuroscience Institute, Methodist Le Bonheur Healthcare, Memphis, TN, United States; 3 Children's Foundation Research Institute, Le Bonheur Children's Hospital, Memphis, TN, United States; 4 Department of Pediatrics, Division of Pediatric Neurology, University of Tennessee Health Science Center, Memphis, TN, United States

**Keywords:** caregivers, education, rescue medication, seizure clusters, YouTube, intranasal, diazepam, midazolam

## Abstract

**Background:**

Patients with seizure clusters and their families often use YouTube videos as resources for information about the illness and how to use concomitant rescue medications; however, many videos include misinformation or incorrect information that could potentially cause harm.

**Objective:**

This study aims to examine YouTube videos intended as educational and instructional resources for seizure clusters and treatment options posted since the approval of intranasal benzodiazepine rescue medications in 2019 and to assess their accuracy and quality using a 3-item rating scale.

**Methods:**

We searched YouTube for 10 specific terms related to seizure clusters and treatment options and analyzed videos from the first 20 results for each to mimic internet user behavior when choosing results with search engines. Videos were rated on 3 criteria: accurate seizure cluster definition, appropriate US Food and Drug Administration (FDA)–approved treatment recommendation, and correct visual demonstration of rescue medication use. A single author rated the videos based on the presence of specific subcriteria of all 3 primary criteria in each video. Score assignment was based on the number of present subcriteria.

**Results:**

Seizure clusters and trade names generated the highest number of videos meeting criteria; other search terms, including nonproprietary names, garnered <8 videos each. Only 19.5% (n=39) of the 200 collected videos provided accurate descriptions of seizure clusters, while 61.0% (n=122) offered no definition, 37.0% (n=74) recommended at least 1 FDA-approved rescue medication, and 37.5% (n=75) offered no rescue therapy at all; 26.5% (n=53) provided a full demonstration of rescue medicine, while 62.5% (n=125) did not. Only 4.5% (n=9) of videos included all subcriteria of the 3 primary criteria. Search results for specific trade names yielded more results, while less specific terminology returned unrelated or unhelpful videos.

**Conclusions:**

Accurate YouTube videos of seizure cluster management are limited and represent a knowledge gap that should be addressed to avoid patient harm. To facilitate better navigation of educational and instructional resources, clinicians should have a ready list of videos available to give to patients or provide a list of search terms that are known to produce accurate results to facilitate better searches. Reputable health care sources could also produce brief, helpful videos of each rescue therapy with search terms that direct patients to their content.

## Introduction

Seizure clusters are often characterized by seizures that deviate from a patient’s normal seizure pattern, appearing intermittently in groups over hours or days [1]. Seizure clusters have been referred to by various names in the past, including seizure flurry, acute repetitive seizures, serial seizures, and crescendo seizures [1-5]. Criteria for seizure clusters range broadly. For example, acute repetitive seizure criteria include experiencing multiple complex partial (now called focal with impaired consciousness) or generalized seizures in 24 hours for adults or 12 hours for children. Additionally, seizure cluster definitions can include experiencing at least 3 seizures in 24 hours [6-11], 3 seizures within 4 hours [8,10,12], or an atypical pattern of seizures that differs from a patient’s usual pattern [12,13]. Despite being recognized as dangerous and potentially fatal [14,15], seizure clusters do not have an established definition [1,16-18]. The International League Against Epilepsy, for example, has yet to establish an official classification. The only available guidance derives from a broad consensus issued by the US Food and Drug Administration (FDA) [1,13] that provides a vague definition of “intermittent, stereotypic episodes of frequent seizure activity (ie, seizure clusters, acute repetitive seizures) that are distinct from a patient’s usual seizure pattern” [19,20].

These different and sometimes conflicting definitions for seizure clusters have led to inconsistent treatment advice with studies providing different recommendations [16-18]. Some recommend only FDA-approved rescue medications—fast-acting medicines given at home to stop seizure clusters—for treating seizure clusters, including rectal diazepam, intranasal diazepam, and intranasal midazolam [21,22]. Nonindicated treatments can include extra doses of maintenance antiseizure medications or oral benzodiazepines [15]. For patients, seizure clusters are challenging to manage without the perplexities of definition and treatment options. The lack of an established definition contributes to the confusion due to the variability of potential triggers. Additionally, patients may experience their own unique set of triggers, or they may not be able to define precise physiologic changes or activities that enhance the likelihood of seizure clusters [13]. Auras may or may not be present to signal the onset of cluster seizures. Being able to recognize seizure clusters and understanding appropriate rescue treatments to prevent and control this acute condition is paramount.

Prior studies have shown that patients and caregivers seek health information from websites, journals, and educational videos, with YouTube being a prominent platform [23,24]. Searches conducted by YouTube users for seizure information suggest a demand for resources; however, the accuracy of the information in these videos is inconsistent. Videos can contain varying levels of accuracy and lack useful information. St-Pierre et al [25] found that 45.3% of the videos assessed in their study focused on personal experience, while 63.5% were considered educational. They also found that 18.1% contained wrong or misleading information. Two raters in Fat et al’s [26] study found 60% and 64%, respectively, of the videos they assessed were accurate, but only 10 videos were given the study’s highest possible rating. Gonulal et al [27] rated 53.3% of the videos in their study as low quality and rated only 1 video as high quality. Wong et al [28] found that most videos (44%) were created by users relating personal experiences or anecdotes, while only 38% were informative or educational. Only 51% of the videos were considered accurate, 40% were neutral, and 9% were inaccurate. Finally, a review by Osman et al [29] concluded overall that YouTube was not a reliable source and that only 32% of produced content was considered unbiased.

Given the lack of classification for seizure clusters and inconsistencies with available treatments, it is no surprise that patients and caregivers may become confused, resort to searching online for information as a result, and heed advice given on social media or found in videos and on websites [14]. While some videos by health care professionals and pharmaceutical companies do include legitimate educational and instructional created content, there is a vast number of sources featuring individuals sharing personal experiences as caregivers and offering what they have learned as sound advice. Most of these videos contain content created by nonmedical professionals. Without expert clinical input to verify information, the advice may be partially or completely incorrect, missing key information, or specific to certain patients and not generalizable. These sources introduce high-risk misinformation that may cause harm.

There are few studies on YouTube videos as a resource for seizure clusters and rescue treatments, many of which do not assess the accuracy of video content. Most studies focus on non–seizure cluster topics. The purpose of this study therefore was to identify and assess the accuracy of YouTube videos intended as educational and instructional resources for seizure clusters and rescue treatments [24,30]. The research question we posed for the study is the following: are the instructional/educational videos on YouTube for seizure clusters and rescue treatments accurate? We hypothesized that most videos analyzed for this study were inaccurate, reflecting a need for improved instruction and education.

## Methods

### Study Design

This was a cross-sectional observational study that evaluated and rated the accuracy of YouTube videos about seizure clusters and treatments. Videos were selected from YouTube based on 3 criteria: seizure clusters definition, rescue therapy indication, and therapy demonstration. Videos were found using 10 specific terms with YouTube’s search feature: (1) *seizure clusters*, (2) *acute repetitive seizures*, (3) *status epilepticus*, (4) *seizure rescue medications*, (5) *rectal benzodiazepines*, (6) *nasal benzodiazepines*, (7) *Nayzilam* (the trade name for intranasal midazolam), (8) *Valtoco* (the trade name for intranasal diazepam), (9) *midazolam*, and (10) *diazepam* [26,28,31]. Searches were performed on YouTube without an account (ie, logged out of YouTube) and in a private browser to avoid potential account-related influences, such as algorithmically determined search results based on an account’s prior search and viewing history.

Searches were conducted by the first author (TSE) between June 2023 and March 2024. Inclusion criteria were videos containing at least 1 search term in the video’s title or description, publication between 2019 and 2024, and inclusion of educational or instructional content. The year 2019 was set as the starting point for search results to include videos created after FDA approval of new intranasal benzodiazepine rescue medications (ie, intranasal midazolam in 2019 and intranasal diazepam in 2020). Exclusion criteria were videos published before 2019, content not specific to seizure clusters or rescue treatments, absence of all 10 search terms in the title or description, or absence of an educational or instructional component, and sponsored videos (ie, videos that appear at the top of search results via paid placement). No filters for language were applied.

Videos from the top 20 search results for each search term were selected for analysis for a total of 200 videos. The cutoff of 20 was intended to mimic how search engine users focus mostly on the first page of results [32]. Specifically, marketing research about search engine user behavior has found that 75% of users never go to the second page of results [33] and that only 21% click on more than 1 result [34], meaning that most users click only 1 link on the first page. Since search terms were closely related and YouTube’s video algorithm recommends videos with similar titles and content, we permitted and included videos that appeared in the search results of multiple search terms. If this occurred, the video was counted once in the overall dataset, but its performance was tabulated under each search term where it appeared.

### Video Analysis

Videos from the top 20 search results for each search term were selected for analysis based on 3 primary criteria mentioned above (seizure cluster definition, treatment recommendation, and demonstration of treatment). Subcriteria for each primary criterion were established by the senior author (an epileptologist). The definition used for seizure clusters was based on the FDA’s broad consensus, as it currently was the only definition available. An accurate definition had to include mention of 2 or more seizures within a 24-hour period. An accurate recommendation for treatment had to include a suggestion to use an FDA-approved medication and a description of the official label indication for appropriate age and route of administration. An accurate video demonstration of how to use the rescue medication had to include images of the device, a verbal discussion of the steps to administer the medication, and an example administration performed with a live person, a dummy, or computer-generated images (Table 1).

**Table 1.** Rating system for YouTube videos.

Primary criteria	Number of subcriteria present	Subcriteria score
Did the video (1) mention and (2) accurately define seizure clusters?	Both	1.0
	One	0.5
	None	0.0
Did the video (1) suggest and (2) discuss indication of Food and Drug Administration–approved rescue medicine?	Both	1.0
	One	0.5
	None	0.0
Did the video (1) include a demonstration of administering the rescue medication and (2) was it accurate?	Both	1.0
	One	0.5
	None	0.0

Videos were assessed for quality with a video rating system developed and validated originally by Fat et al [26] in their study evaluating YouTube videos as educational tools for infantile spasms. The present study adapted this system by excluding technical quality criteria originally set by Fat et al [26] (ie, quality of lighting, sound, etc) and focusing solely on topical quality based on the primary criteria for seizure clusters in place of infantile spasm criteria.

For the present study, the 3 seizure cluster criteria were divided into 2 subcriteria (Table 1). Scoring was based on the presence of both, one, or none of the subcriteria for each primary criterion. Scores for each video were then tallied, reflecting the number of elements present and correctly conveyed. Overall video scores ranged between a minimum score of 0 to a maximum score of 3, which was an author-developed pragmatic tool rather than a validated instrument. Cutoffs were chosen a priori by the authors to distinguish videos that omitted the most critical elements from those that provided comprehensive instruction. Levels of quality were rated with the following ranges of overall scores: 0.0‐0.5=very poor quality, 1.0‐1.5=poor quality, 2.0‐2.5=good quality, and 3.0=great quality. The first author (TSE) conducted all video ratings.

### Ethical Considerations

This study did not include any protected health information from electronic medical records, so an institutional review board approval was not required. Any personal information related to the videos or YouTube channels discussed in the manuscript was deidentified for the study.

## Results

### Search Results for Total Sample

A total of 200 videos were collected for study with 20 videos per search term. Video posting dates ranged from 2019 to 2024 (Table 2). Mean overall length of all 200 videos was 13.5 (SD 19.0, range 0.3‐120) minutes long. The longest length by search term was *acute repetitive seizures* (mean 37.4, SD 38.7, range 1‐120), followed by *status epilepticus* (mean 22.1, SD 24.8, range 4‐100) and *seizure clusters* (mean 12.7, SD 14.8, range 0.5‐52). Videos retrieved from the search term *nayzilam* had the shortest video length (mean 4.2, SD 3.0, range 1‐14). Most videos were presented/narrated by or based their authority on the credentials of a medical doctor (n=34, 17.0%), hospital (n=37, 13.5%), or company (n=23, 11.5%). Some videos included combined authority, such as a medical doctor in combination with a hospital (n=12, 6.0%), organization (n=6, 3.0%), company (n=3, 1.5%), or medical school (n=3, 1.5%). The YouTube channels with the greatest numbers of posted videos were Neurelis (n=20, 10.0%), Valley Children’s (n=19, 9.5%), Epilepsy Foundation (n=16, 8.0%), and UCB (n=9, 4.5%). Eighty-two channels posted only a single video.

**Table 2.** Characteristics of sampled videos (N=200).

Characteristic	Value
Earliest year of posting	2019
Latest year of posting	2024
Video duration for total sample (minutes), mean (SD, range)	12.5 (19.0, 0.3‐120)
Video duration by search term (minutes), mean (SD, range)	
Acute repetitive seizures (n=20)	37.4 (38.7, 1-120)
Status epilepticus (n=20)	22.1 (24.8, 4-100)
Seizure clusters (n=20)	12.7 (14.8, 0.5‐52)
Valtoco (n=20)	11.6 (11.9, 3-45)
Seizure rescue medications (n=20)	12.3 (13.2, 2-52)
Diazepam (n=20)	7.7 (7.0, 2-29)
Rectal benzodiazepines (n=20)	7.5 (5.2, 1-20)
Nasal benzodiazepines (n=20)	6.3 (2.2, 0.3‐20)
Midazolam (n=20)	3.7 (2.2, 2-29)
Nayzilam (n=20)	4.2 (3.0, 1-14)
Video by presenter and credential, n (%)	
Medical doctor	34 (17.0)
Hospital	27 (13.5)
Company	23 (11.5)
Organization	18 (9.0)
Registered nurse	15 (7.5)
None	14 (7.0)
Medical doctor, hospital	12 (6.0)
Doctor of pharmacy	6 (3.0)
Medical doctor, organization	6 (3.0)
Registered nurse, company	5 (2.5)
Patient	4 (2.0)
Patient, company	4 (2.0)
Registered pharmacist	4 (2.0)
Medical doctor, company	3 (1.5)
Medical doctor, medical school	3 (1.5)
Patient, parent, company	3 (1.5)
Emergency medical technician	2 (1.0)
Medical doctor (psychiatrist)	2 (1.0)
News source	2 (1.0)
Patient, parent	2 (1.0)
Unconfirmed/possible medical doctor	2 (1.0)
Doctor of pharmacy, company	1 (0.5)
Medical doctor, hospital, organization	1 (0.5)
Medical doctor, news source	1 (0.5)
Medical doctor (general practitioner)	1 (0.5)
Parent, organization	1 (0.5)
Physician assistant, certified	1 (0.5)
Registered nurse, organization	1 (0.5)
Veterinary medical doctor	1 (0.5)
Veterinary medical doctor, veterinary school	1 (0.5)
Videos posted by channel name, n (%)	
Neurelis	20 (10.0)
Valley Children’s	19 (9.5)
Epilepsy Foundation	16 (8.0)
UCB	9 (4.5)
Neurology Live	6 (3.0)
Drug Talk	4 (2.0)
Cleveland Clinic	3 (1.5)
CURE Epilepsy	3 (1.5)
Orlando Health	3 (1.5)
VJNeurology	3 (1.5)
Andrew Kim MD	2 (1.0)
Child Neurology Foundation	2 (1.0)
drbillakotamd	2 (1.0)
ILAE - International League Against Epilepsy	2 (1.0)
Johns Hopkins Medicine	2 (1.0)
Just a Different Life	2 (1.0)
Medical Videos [Animated]	2 (1.0)
Medicating Normal	2 (1.0)
Medzcool	2 (1.0)
Mini Med Lessons	2 (1.0)
Nurse Ryan	2 (1.0)
Paediatric Emergencies	2 (1.0)
Pixorize	2 (1.0)
RegisteredNurseRN	2 (1.0)
Seattle Children’s	2 (1.0)
Strong Medicine	2 (1.0)
Channels with only 1 (0.5%) video each	82 (41.0)

Videos from the top 20 search results for each search term included 103 (51.5%) total videos scored as very poor, 24 (12.0%) scored as poor, 51 (25.5%) scored as good, and 22 (11.0%) scored as great (Table 3). The search term that retrieved the highest scoring videos was *valtoco* (n=20, 10.0%), which garnered 3 great, 15 good, 2 poor, and 0 very poor ratings. Conversely, the search term that retrieved the lowest scoring videos was *status epilepticus* with 20 (10.0%) videos rated very poor. Only 9 (4.5%) distinct videos in the total sample met all 3 inclusion criteria. (Links to these videos are provided in Multimedia Appendix 1.)

**Table 3.** Videos by search term ratings (N=200).

Total sample and search term	Very poor, n (%)	Poor, n (%)	Good, n (%)	Great, n (%)
All videos (N=200)	103 (51.5)	24 (12.0)	51 (25.5)	22 (11.0)
Acute repetitive seizures (n=20)	11 (5.5)	3 (1.5)	6 (3.0)	0 (0.0)
Diazepam (n=20)	17 (8.5)	0 (0.0)	0 (0.0)	3 (1.5)
Midazolam (n=20)	15 (7.5)	1 (0.5)	3 (1.5)	1 (0.5)
Nasal benzodiazepines (n=20)	12 (6.0)	2 (1.0)	4 (2.0)	2 (1.0)
Nayzilam (n=20)	3 (1.5)	5 (2.5)	9 (4.5)	3 (1.5)
Rectal benzodiazepines (n=20)	16 (8.0)	0 (0.0)	2 (1.0)	2 (1.0)
Seizure clusters (n=20)	4 (2.0)	5 (2.5)	6 (3.0)	5 (2.5)
Seizure rescue medications (n=20)	5 (2.5)	6 (3.0)	6 (3.0)	3 (1.5)
Status epilepticus (n=20)	20 (1.0)	0 (0.0)	0 (0.0)	0 (0.0)
Valtoco (n=20)	0 (0.0)	2 (1.0)	15 (7.5)	3 (1.5)

Analysis of primary criteria and subcriteria found that only 39 (19.5%) videos provided accurate descriptions of seizure clusters, 39 (19.5%) defined seizure clusters partially, and 122 (61.0%) offered no definition at all (Table 4). Of the total sample, 74 (37.0%) videos recommended at least 1 FDA-approved rescue medication, 32 (16.0%) suggested an unapproved medication for seizure emergencies, 19 (9.5%) discussed both approved and unapproved medications, and 75 (37.5%) offered no rescue therapy at all. Of the 74 videos that did recommend an FDA-approved medication, 69 (93.2%) fully and accurately described its indication, while 3 (4.05%) only offered a partial discussion of rescue indication and 2 (2.7%) included none. Of the total sample, 53 videos (26.5%) provided a full demonstration of rescue medicine, 22 (11.0%) provided an incomplete demonstration, and 125 (62.5%) did not provide any demonstration. Of the combined 75 videos that provided either a full or partial demonstration of the medication, 16 (21.3%) showcased the device alone, 5 (6.67%) included a verbal description of its administration, 6 (8.0%) included both, and 49 (65.3%) provided a guided demonstration with a human being, a dummy, or computer-generated images.

**Table 4.** Analysis of video quality criteria based on search results of each search term (N=200).

Total sample and search term	Seizure clusters mentioned, n (%)		Definition given in videos that mentioned seizure clusters, n (%)^a^		Rescue medications discussed, n (%)				Medicine indication(s) discussed/defined, n (%)			Medicine administration demonstrated, n (%)		
	Yes	No	Full and accurate	Incomplete or inaccurate	FDA^b^-approved	Non–FDA-approved	Both	None	Full	Partial	None	Full and accurate	Incomplete or inaccurate	None
All videos (N=200)	78 (39.0)	122 (61.0)	39/78 (50.0)	39/78 (50.0)	74 (37.0)	17 (8.5)	16 (8.0)	93 (46.5)	69 (34.5)	3 (1.5)	128 (64.0)	52 (26.0)	23 (11.5)	125 (62.5)
Seizure clusters (n=20)	15 (75.0)	5 (25.0)	10/15 (66.7)	5/15 (33.3)	9 (45.0)	2 (10.0)	4 (20.0)	5 (25.0)	12 (60.0)	1 (5.0)	7 (35.0)	6 (30.0)	1 (5.0)	13 (65.0)
Acute repetitive seizures (n=20)	8 (40.0)	12 (60.0)	4/8 (50.0)	4/8 (50.0)	3 (15.0)	5 (25.0)	4 (20.0)	8 (40.0)	4 (20.0)	1 (5.0)	15 (75.0)	1 (5.0)	6 (30.0)	13 (65.0)
Status epilepticus (n=20)	0 (0.0)	20 (100.0)	0/0 (0.0)	0/0 (0.0)	0 (0.0)	0 (0.0)	0 (0.0)	20 (100.0)	0 (0.0)	0 (0.0)	20 (100)	0 (0.0)	0 (0.0)	20 (100.0)
Seizure rescue medications (n=20)	12 (60.0)	8 (40.0)	5/12 (41.7)	7/12 (58.3)	8 (40.0)	4 (20.0)	5 (25.0)	3 (15.0)	12 (60.0)	1 (5.0)	7 (35.0)	9 (45.0)	5 (25.0)	6 (30.0)
Rectal benzodiazepines (n=20)	3 (15.0)	17 (85.0)	3/3 (100.0)	0/3 (0.0)	4 (20.0)	5 (25.0)	0 (0.0)	11 (55.0)	3 (15.0)	0 (0.0)	17 (85.0)	3 (15.0)	0 (0.0)	17 (85.0)
Nasal benzodiazepines (n=20)	6 (30.0)	14 (70.0)	2/6 (33.3)	4/6 (66.7)	8 (40.0)	0 (0.0)	0 (0.0)	12 (60.0)	5 (25.0)	0 (0.0)	15 (75.0)	6 (30.0)	0 (0.0)	14 (70.0)
Nayzilam (n=20)	9 (45.0)	11 (55.0)	3/9 (33.3)	6/9 (66.7)	15 (75.0)	0 (0.0)	2 (10.0)	3 (15.0)	11 (55.0)	0 (0.0)	9 (45.0)	12 (60.0)	1 (5.0)	7 (35.0)
Valtoco (n=20)	18 (90.0)	2 (10.0)	8/18 (44.4)	10/18 (55.6)	19 (95.0)	0 (0.0)	1 (5.0)	0 (0.0)	18 (90.0)	0 (0.0)	0 (0.0)	9 (45.0)	8 (40.0)	3 (15.0)
Midazolam (n=20)	3 (15.0)	17 (85.0)	1/3 (33.3)	2/3 (66.7)	5 (25.0)	1 (5.0)	0 (0.0)	14 (70.0)	2 (10.0)	0 (0.0)	18 (90.0)	4 (20.0)	1 (5.0)	15 (75.0)
Diazepam (n=20)	4 (20.0)	16 (80.0)	3/4 (75.0)	1/4 (25.0)	3 (15.0)	0 (0.0)	0 (0.0)	17 (85.0)	2 (10.0)	0 (0.0)	18 (90.0)	2 (10.0)	1 (5.0)	1 (85.0)

a Denominator varies by search term based on the number of videos that mentioned seizure clusters and is indicated in each cell.

b FDA: US Food and Drug Administration.

### Search Results by Term

Across search terms, there was variability in terms of content, discussion, and demonstration (Table 4 and Table 5). Direct and clinically relevant searches with terms such as *seizure clusters*, *seizure rescue medications*, *Valtoco*, and *Nayzilam* were more likely to return videos with higher accuracy and better demonstrations (ie, most or all subcriteria of the primary criteria were met). For example, results for the term *seizure clusters* included 5 (25.0%) great and 6 (20.0%) good videos. Results for the term *seizure rescue medications* included 3 (15.0%) great and 6 (30.0%) good videos. Results for the term *Valtoco* included 3 (15.0%) great and 15 (75.0%) good videos. Results for the term *Nayzilam* included 3 great (15.0%) and 9 good (45.0%) videos.

**Table 5.** Analysis of the type of administration demonstration by search term.

Search term (n=20 for each)	Images only, n (%)	Verbal description only, n (%)	Images and verbal description, n (%)	Full demonstration presented^a^, n (%)
Seizure clusters	1 (5.0)	—^a^	4 (20.0)	2 (10.0)
Acute repetitive seizures	5 (25.0)	—	1 (5.0)	—
Status epilepticus	—	—	—	—
Seizure rescue medications	1 (5.0)	3 (15.0)	—	10 (50.0)
Rectal benzodiazepines	—	—	—	3 (15.0)
Nasal benzodiazepines	—	—	—	6 (30.0)
Nayzilam	1 (5.0)	—	—	12 (60.0)
Valtoco	8 (40.0)	—	—	9 (45.0)
Midazolam	—	—	—	4 (20.0)
Diazepam	—	—	—	3 (15.0)

a Demonstration performed with an individual, a dummy, or computer-generated images.

b Not applicable.

Conversely, searching for generic medication names (*midazolam* and *diazepam*) or their drug class (*benzodiazepines*) resulted in videos with lower quality grades. Specifically, results for the term *midazolam* returned only 3 (15.0%) videos that discussed seizure clusters, 2 (10.0%) provided information about indications for treatment, and 4 (20.0%) included a demonstration of medication use, while 14 (70.0%) discussed incorrect therapies or no therapies at all. Results for the term *diazepam* generated similar results, with only 4 (20.0%) videos providing a definition for seizure clusters, 17 (85.0%) videos lacking discussions of rescue medications, 18 (90.0%) lacking indications to treat seizure clusters, and 17 (85.0%) lacking demonstrations. Results for the terms *rectal benzodiazepines* and *nasal benzodiazepines* were similar.

Terms associated with seizure clusters—*acute repetitive seizures* and *status epilepticus—*yielded few videos for patient and health care provider education. Searching for *acute repetitive seizures* resulted in 8 (40.0%) videos that mentioned seizure clusters but only 3 (15.0%) described their management with rescue medication, 4 (20.0%) discussed why they should be treated, and only 1 (5.0%) offered visualizations of how to use appropriate therapies. Interestingly, across all metrics, *status epilepticus* failed to provide any videos that included a seizure cluster description, suggested treatment option, and demonstration of medication.

## Discussion

### Principal Findings

Given the unpredictability of seizure clusters, their difference from normal seizure patterns, and their higher risk for injury and sudden unexpected death in epilepsy, effective instruction for administering rescue medications is vital. This analysis determined that even while using highly specific terminology related to seizure emergencies and rescue therapies, most YouTube videos collected were rated as poor or very poor (combined n=127, 63.5%). By comparison, only 25.5% (n=51) of videos were rated as good, and 11.0% (n=22) were rated as great. *Seizure clusters*, *Nayzilam*, and *Valtoco* were the only terms in which the number of effective videos found exceeded the number of ineffective videos and were more likely to be seen by medical professionals, possibly because of their link to medical jargon. The results from this study showed that specificity is key to navigating YouTube, and that search terms directly linked to seizure clusters and approved treatments were more effective for gathering accurate videos.

One of the principal findings of this study was that a large proportion of videos contained missing information. While 19.5% (n=39) of the videos provided accurate descriptions of seizure clusters and another 19.5% (n=39) provided a partial or inaccurate description, most videos (n=122, 61.0%) included no definition at all. This pattern also repeated with the other primary criteria. Only 37.0% (n=74) of the videos provided information about FDA-approved treatments, and 37.5% (n=75) did not provide any recommended treatments. While 34.5% (n=69) of the videos included the full indication for the rescue treatment and 1.5% (n=3) included a partial indication, 64.0% (n=128) included none. Only 26.0% (n=52) provided a complete demonstration of the therapy and 11.5% (n=23) provided an incomplete demonstration, and 62.5% (n=125) provided none. These findings highlight a wide gap in knowledge about seizure clusters in video resources on YouTube. Patients with seizure clusters do not have many video resources to choose from. If they found videos with missing information, it is likely that they would not be able to recognize that this information was absent.

Most of the videos with high ratings provided either sufficient details about rescue indication, purpose, and pharmacology, or they provided a well-organized and thorough procedural guide for administration of the medication—few achieved both. As search terms deviated from seizure clusters toward other seizure emergencies and their treatment, including *status epilepticus* and *rectal benzodiazepines*, the quality of videos decreased. Similarly, as expected, using more general search terminology retrieved videos with less applicability to seizure clusters and rescue medications. These types of search results could be problematic for anyone looking for accurate information. Patients and their caregivers would need to sift through videos with missing information, and roughly half of the videos that did include information could be wrong.

### Literature

There are few papers in the literature that have analyzed YouTube video content and seizure clusters. A 2026 study of 563 total videos across multiple platforms, including YouTube, by St-Pierre et al [25] found that 45.3% of the total videos focused on personal experience and 18.1% contained wrong or misleading information. The majority of YouTube videos were labeled as educational (63.5%), but only 8% were considered to be inaccurate. Fat et al [26] assessed 28 videos intended as a resource for patients with infantile spasms, and 2 raters found 60% and 64% of videos to be accurate, respectively, with only 10 videos rated as excellent. Unlike Osman et al [29], the authors concluded that YouTube could be a beneficial patient resource with guided searches; however, Fat et al [26] used a methodology that included technical quality in their rating system, which composed 5 out of 7 criteria and comprised 45% of the total score. Only 1 criterion worth 1 point was used for diagnostic accuracy, and 1 criterion worth 5 points was used to rate the overall quality of the video. As a result, videos could have high scores due to excellent video production and presentation. It is possible that most of the scores were thus based on superficial video and style qualities, and that a more careful assessment of health information content would yield results closer in line with Osman et al [29]. Gonulal et al [27] published a 2026 systematic analysis of YouTube videos for epilepsy-specific exercises and found that 53.3% of the 45 total videos were rated as low quality and only 1 video rated as high quality. Wong et al [28] studied the presentation of seizures and epilepsy in 100 YouTube videos and found that most videos (44%) were created by users relating personal experiences or anecdotes, while only 38% were informative or educational. Only 51% of the videos were considered accurate, 40% were neutral, and 9% were inaccurate.

A recent 2026 cross-sectional analysis by Alpua and Yoldaş [35] assessed the top 50 search results for keywords related to functional neurological disorder. Using several quality scales (Global Quality Scale, modified DISCERN, and *Journal of American Medical Association* benchmark criteria), the study assessed the sample as having “moderate” quality overall but “suboptimal adherence to health information standards.” Analysis of user engagement found a strong correlation between view and like count but a limited association with information quality. A 2020 systematic literature review of 202 studies by Osman et al [29] examined medical and health information in general presented in YouTube videos and concluded that YouTube was not a reliable source. Only 32% of the 22,300 videos analyzed produced content the authors considered unbiased. The authors also reported that videos retrieved in search results were likely selected based on popularity (the number of views and likes) as opposed to any metrics about content. Using this type of methodology suggests that videos with high production values but incorrect information could be driven to the top of search results based solely on engagement. In their study of YouTube videos for functional neurological disorder education, Alpua and Yoldaş [35] emphasized this view by pointedly noting that “engagement metrics do not reliably reflect informational accuracy.”

While YouTube and video demonstrations have their advantages, misinformation is a concern. Content posted by nonmedical professionals was highly variable and often rated as poor, while videos of recorded virtual conferences or of organization-based rescue therapy guides were more likely to be rated as effective due to the inclusion of medical expertise [30,31]. When seizure clusters are the primary concern, understanding their nuances and personalizing therapy is paramount to achieving optimal outcomes; therefore, general videos about epilepsy or seizure therapies may not be ideal for guiding nonmedical professionals, underpinning the need for highly specific searches or direct links to high-quality videos [13,15,22].

Despite nearly half (n=98, 49.0%) of the videos in our study being narrated and published by health care professionals and organizations, there was still a high degree of variability in seizure cluster definition and treatment recommendation. Videos made by actual patients and caregivers appeared more focused on sharing their experience than educating others about seizure management. During instances of seizure emergencies, caregivers in need of a concise, step-by-step tutorial for rescue medications may find incomplete or incorrect information. Additionally, inefficient search terms could further hinder this process. Thus, recommended videos, search terms, and other informational resources for patients and caregivers should be integrated into patient-physician conversations to assist with education and answer lingering questions. Providing this information could prevent patients from accessing inaccurate information, as well as promoting trust in the physician-patient relationship. It could also reduce potential confusion regarding signs and treatment of seizure clusters and empower caregivers to research and administer rescue therapy themselves.

Because of the variability of accuracy and quality of videos found for this study, clinicians should provide specific search terms and direct links to vetted videos for families. Epilepsy centers and professional organizations could also produce their own short, standardized demonstration videos aligned with product labels. There is an opportunity for hospital systems to create curated playlists by audience (eg, families, school nurses, health care providers), as already done by some groups.

### Limitations

There are several limitations associated with this study. A major methodological limitation includes the time window in which searches were conducted. Searches were not done at the same time but rather spread out over months, meaning that there is a high degree of variability in the results. Other limitations include restricting the number of videos to the first 20 results, including repeated videos from different search terms, and providing no analysis or survey of actual patient feedback about video quality to determine video usefulness. The study also used a single rater, thus causing potential selection bias, which is likely to be present in the results, and a potential issue with rater reliability. Additionally, this study relied entirely on YouTube’s search function, which is itself subject to various and unknown proprietary internal algorithms for gathering results. This concern is especially amplified considering the increasing use of AI among tech companies. Despite this study’s efforts to limit algorithmic influence from YouTube, there is no guarantee that results were completely unbiased. Finally, the fluidity of online content, which is in a constant state of change, means that future studies are unlikely to replicate our results.

### Conclusions

Seizure rescue medications are vital to halting seizure clusters, but seizure rescue medications can be challenging when their use and purpose are not properly explained or demonstrated. This study found that most videos posted between 2019 and 2024 within the top 20 search results for 10 seizure-related terms included missing information or lacked key elements necessary for safe and effective rescue use. Many videos were inconsistent in the information conveyed, and some included incorrect or wrongly interpreted information. The stark contrast found in the search results among different search terms indicates a gap in accessibility of educational tools that should be highlighted and remedied by health care professionals.

Being able to differentiate seizure clusters from status epilepticus and recognizing appropriate rescue treatments to prevent and control these acute conditions is paramount and further underscores the gap in accessible information for the public. In the short term, clinicians should have a ready list of effective YouTube videos to give patients during outpatient and inpatient discussions, provided in a card, brochure, or document that includes links to the most appropriate videos. Alternatively, clinicians could provide specific search terms—such as *seizure rescue medications* or proprietary product names—that are known to return useful and accurate videos. Finally, health care professionals and organizations could create their own videos with easily identifiable information, such as hospital or clinic names, and use targeted search engine optimization strategies that could increase the likelihood that patients are directed to their content and away from inaccurate content.

Future studies on this topic should include the development and validation of an instrument for standardizing video quality (one with the potential to be generalizable to other studies of social media content beyond seizure clusters). Other potential avenues for research include examinations of search results from the years following this study’s cutoff, quantitative analysis of viewer data (eg, counts of views, likes, and comments), and qualitative analysis of comment text to determine most-used videos and their accuracy. Finally, longitudinal monitoring of content quality over time could also enable health care providers to locate gaps and assist in creating media content with current up-to-date seizure cluster treatment information.

## Supplementary material

10.2196/98768 Multimedia Appendix 1 Video links to highest quality videos.

## Abbreviations

FDA: Food and Drug Administration
