# Supplementary material for: YouTube Videos as Educational Resources for Seizure Clusters and Treatment Options: Cross-Sectional Observational Study
Source: JMIR Infodemiology. 2026 Sep 30;6:e98768. doi: 10.2196/98768 (PMC13627083; doi:10.2196/98768)
Supplement: Supplementary file 1 [file infodemiology-v6-e98768-s001.docx]

Multimedia Appendix

| **Instructional YouTube Videos Meeting all Study Criteria.** | | |
| --- | --- | --- |
| **Title** | **Channel** | **Link** |
| Diastat Training | Valley Children's | https://youtu.be/AYPzm4xdOqQ?si=YKqBahuQC9l5K31S |
| Diastat Training (Español) | Valley Children's | https://youtu.be/yioLPwQrcEY?si=75jt2hgFwxerrPeq |
| Epilepsy Emergencies and Seizure Rescue Medications | CURE Epilepsy | https://youtu.be/x_tYDpOU0jI?si=UMMXM6X5uk658Pm0 |
| Living with Clusters and Rescue Medication | Purple Day Every Day (Anita Kauffman Foundation) | https://youtu.be/4iwQMFxJHbM?si=2CJtPvu3z2bK_Oph |
| Nayzilam Training | Valley Children's | https://youtu.be/L-591DbR0Rg?si=dzSKqQUl0ecRZ0ft |
| Nayzilam Training (Español) | Valley Children's | https://youtu.be/uBmRRXakDAY?si=ppxrKF1xr_dI1w2K |
| Treating Seizure Clusters with Midazolam | Neurology Live | https://youtu.be/DAA75On8D9c?si=Z0sDwA_7zNjQOljc |
| Valtoco Training | Valley Children's | https://youtu.be/YfKsPG6P-2Y?si=5FesaOWdYe_PNkZv |
| Valtoco Training (Español) | Valley Children’s | https://youtu.be/T-7L0QllXJM?si=Egk4-89RBHh0ZXN2 |
